# Immediate postoperative topical lidocaine gel for the treatment of eye pain following corneal abrasion in descemet membrane endothelial keratoplasty (DMEK) under general anaesthesia: a pilot retrospective analysis

**DOI:** 10.1186/s12871-023-02258-y

**Published:** 2023-09-09

**Authors:** Nicolas Leister, Björn Bachmann, Mario Matthaei, Uwe Trieschmann, Christine Schumacher, Vanessa Löw, Bernd W. Böttiger, Silvia Schrittenlocher, Ludwig M. Heindl, Claus Cursiefen

**Affiliations:** 1 Department of Anesthesiology and Intensive Care Medicine, University Hospital Cologne, Kerpener Street 62, 50937 Cologne, Germany; 2 Department of Ophthalmology, Faculty of Medicine, University of Cologne, University Hospital Cologne, Cologne, Germany

**Keywords:** Postoperative pain, Topical lidocaine gel, Descemet membrane endothelial keratoplasty, Iatrogenic corneal abrasion

## Abstract

**Background:**

Patients undergoing corneal abrasion as part of Descemet membrane endothelial keratoplasty (DMEK) under general anesthesia suffer from early burning pain postoperatively. This pain appears to be poorly treatable with systemic analgesics. This study aims to evaluate postoperative pain management using topical lidocaine gel after DMEK with iatrogenic corneal abrasion.

**Methods:**

Retrospective analysis of 28 consecutive patients undergoing DMEK with corneal abrasion from October 19, 2021, to November 12, 2021, at a German university hospital. Patients during week 1 and 2 received peri-operative standard pain treatment (cohort S) and additional local lidocaine gel during week 3 and 4 immediately postoperatively (cohort L).

**Results:**

13 patients were included in cohort S and 15 patients in cohort L. At awakening all patients (100%) in cohort S reported burning pain, and six of 15 patients (40%) in cohort L reported burning pain. Burning pain scores were significantly lower in cohort L (p < 0.001 at awakening, p < 0.001 at 10 min, p < 0.001 at 20 min, p < 0.001 at 30 min, p = 0.007 at 40 min after awakening, and p < 0.001 at leaving recovery room). No significant differences between cohort S and cohort L were detected concerning surgical outcome during 1-month-follow-up (p = 0.901 for best corrected visual acuity).

**Conclusion:**

Patients undergoing DMEK with corneal abrasion suffer significant pain in the recovery room. A single dose of topic lidocaine gel reduces the early postoperative burning pain sufficiently and does not affect the surgical outcome.

## Key messages

*What is already known on this topic*:

Descemet membrane endothelial keratoplasty (DMEK) normally causes low levels of pain.

*What this study adds*:

When corneal abrasion is required during DMEK surgery under general anesthesia, patients experience pain sensations that can severely affect their well-being.

An immediate postoperative single dose of topical lidocaine gel applied by the surgeon reduces the early burning pain sufficiently and does not affect the surgical outcome.

To reduce postoperative pain levels and to improve the postoperative well-being of patients, all patients undergoing DMEK with corneal abrasion should receive topical lidocaine gel at least once. However, this simple intervention should still be investigated in prospective studies.

## Introduction

Descemet membrane endothelial keratoplasty (DMEK) is a standardized therapy for corneal endothelial diseases. [1] DMEK can be performed under general or regional/local anesthesia. Functional results seem to be comparable. [2] DMEK for endothelial pathologies (e.g., pseudophakic bullous keratopathy or Fuchs endothelial corneal dystrophy) is historically described as being associated with low postoperative pain intensity. Elevated pain levels have been associated with elevated intraocular pressure in recent literature. [3] Early postoperative pain is common and reduces the patient´s well-being, furthermore it may lead to persistent or chronic postoperative pain. [4, 5] Although postoperative pain intensity is described to be low in patients undergoing DMEK, the authors identified a subgroup with extensive burning pain sensations in recovery room: patients with the need for iatrogenic corneal abrasion during DMEK under general anesthesia. As the majority of patients undergo general anesthesia for DMEK in the authors´ center, for standard treatment systemic analgesia (opioid/non-opioid-combination) was used. The burning pain of patients with corneal abrasions appeared to be practically untreatable with this approach, well-being was sustainably impaired during recovery room stay. To improve these patients´ welfare and reduce their burning pain sensation the effect of topical lidocaine gel applied by the surgeon at the end of surgery was evaluated.

## Materials and methods

### Ethics committee approval

The responsible ethics committee (No: 22-1025) of University Hospital of Cologne, Cologne, Germany approved the study and, also waived the requirement to obtain informed consent from patients to review and use the data. The medical records of all patients who underwent DMEK in general anesthesia from October 19, 2021, to November 12, 2021 were reviewed. The study was conducted in accordance with the Helsinki Declaration on Patient Safety in Anesthesiology. [6].

### Patient and data selection – including criteria

All patients (twenty-eight) undergoing DMEK with the need for corneal abrasion in general anesthesia during October 19, 2021, to November 12, 2021 were enrolled in this retrospective study. During the initial 2-week period, all patients who underwent DMEK received systemic opioid/non-opioid combination therapy as usual for postoperative pain management (cohort S). During the following 2-week-period, patient´s pain after DMEK was treated with this standard combination and additional local lidocaine gel (Xylocain Gel 2%; Aspen Pharma Trading Limited; Dublin, Ireland) administered by the surgeon at the end of surgery (cohort L). The relevant data on pain scores were recorded by hand in standardized anesthesia protocols. 1-month-follow-up examination was documented in standard patient record. Later, the medical record was transferred to the digital patient record via scan. These data were retrospectively reviewed and evaluated for quality assessment of the additive pain therapy intervention.

### Performing the general anesthesia, surgery, and pain therapy

All patients underwent general anesthesia with propofol (2–3 mg/kg bodyweight) and remifentanil (1 µg/kg bodyweight) for induction and sevoflurane (up to 1 MAC) and remifentanil (0.1–0.3 µg/kg/min) for maintenance. The airway was established with a laryngeal mask (second generation) in all patients. All patients received dexamethasone 4 mg and granisetron 1 mg. Patients received piritramide intravenously 10 min after induction of anesthesia (for piritramide dosage, see the “Results” section), and in the absence of a contraindication, they received additional parecoxibe 40 mg. DMEK was performed under deep general anesthesia as described previously (7). In all patients around 5 mm epithelium was removed centrally. The opacified epithelium was removed using a hockey knife to enhance visibility during surgery. No exact measurement was done at time of scraping. In group L, the surgeon applied 0.5 cm of lidocaine gel to the surface of the affected eye at the end of surgery. Furthermore, remifentanil was discontinued, the laryngeal mask was removed, and the patient was transferred to the recovery room. After awakening and every 10 min in the recovery room, pain scores (NRS, numerical rating scale; VRS, verbal rating scale) were measured by the nurse in charge. Before discharge to normal ward, pain scores were measured by the attending physician in charge and the nurse. A numeric rating scale (NRS; 0–10; none to severe) was used to evaluate overall pain, and a verbal rating scale (VRS; 0–3; none to severe) was used to evaluate burning pain. One month after surgery, a follow-up examination regarding ophthalmologic outcome was routinely performed.

### Statistical analysis

The statistical analysis was carried out using SPSS version 27.0 (IBM, SPSS Statistics, IBM Corporation, Chicago, IL, USA).

Distribution of demographic data (age, weight etc.) is presented as mean (95%-confidence interval), since there was no normal distribution. Further data are given as mean (+/- standard deviation) and mean (+/- standard error of mean). Descriptive data are given as numbers (proportion). ANOVA analyses and Mann-Whitney-U-Test were performed to test for significant changes in pain sensations and in opioid use. All p values below 0.05 were considered statistically significant.

## Results

### Demographic data - cohorts

Between October 19, 2021, and November 12, 2021 a total of 28 patients underwent DMEK with corneal abrasion in general anesthesia. 13 patients were included in cohort S and 15 patients in cohort L. The mean age in cohort S was 65.4 years (range, 58.3–72.5 years), body weight was in mean 91.8 kg (range, 77.3–106.3 kg). Cohort L had a mean age of 66.4 years (range, 58.4–74.4 years) and a mean body weight of 89.4 kg (range, 75.5–103.3 kg). Surgical procedures lasted between 25 and 42 min, with no significant difference between cohorts.

## Pain

Before surgery, all included patients denied pain in the affected eye.

All patients in cohort S reported burning pain at awakening with maximum VRS of 3. Six of 15 patients (40%) in cohort L reported burning pain, the maximum was VRS 2. Pain scores see Tables 1 and 2.

**Fig. 1:**
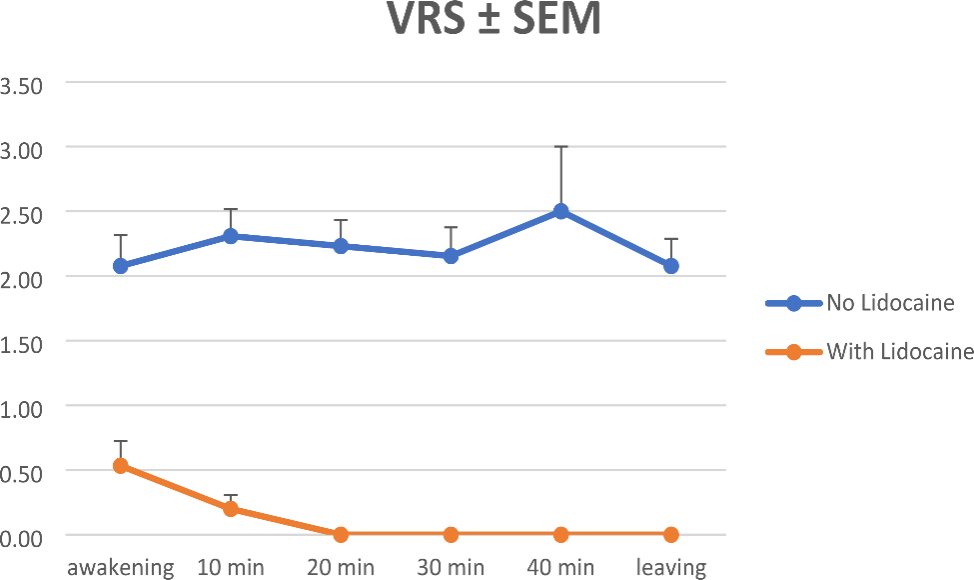
Burning pain VRS scores. VRS, verbal rating scale; SEM, standard error mean;

**Fig. 2:**
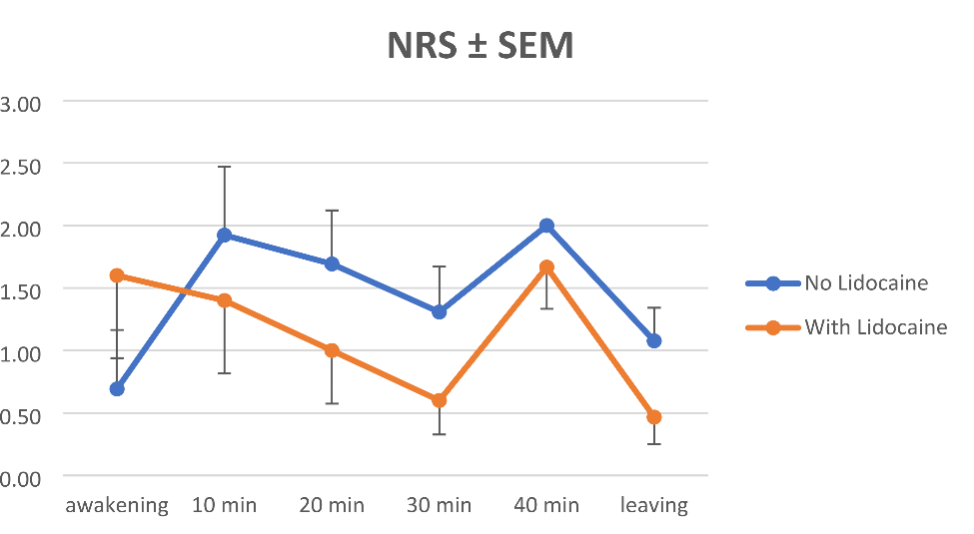
Pain NRS scores. NRS, numerical rating scale; SEM, standard error mean;

Using ANOVA analyses measured burning pain scores were significantly lower in cohort L (p < 0.001 at awakening, p < 0.001 at 10 min, p < 0.001 at 20 min, p < 0.001 at 30 min, p = 0.007 at 40 min after awakening and p < 0.001 at leaving recovery room; see Fig. 1). Mann-Whitney-U-Test showed no significance at 40 min p = 0.053. However, only 5 patients (2 from cohort S/3 from cohort L) remained until this time point. NRS showed no significant difference (p = 0.287 at awakening, p = 0.524 at 10 min, p = 0.265 at 20 min, p = 0.126 at 30 min, p = 0.495 at 40 min after awakening and p = 0.082 at leaving recovery room; see Fig. 2).

**Table 1:** Pain scores (NRS) in cohorts

	NRS Awake			NRS 10 min			NRS 20 min			NRS 30 min			NRS 40 min			NRS leaving		
cohort	S	L	Overall	S	L	Overall	S	L	Overall	S	L	Overall	S	L	Overall	S	L	Overall
N	13	15	28	13	15	28	13	15	28	13	15	28	2	3	5	13	15	28
mean	0,69	1,6	1,18	1,92	1,4	1,64	1,69	1	1,32	1,31	0,6	0,93	2	1,67	1,8	1,08	0,47	0,75
SD	1,702	2,558	2,212	1,977	2,261	2,112	1,548	1,648	1,611	1,316	1,056	1,215	0	0,577	0,447	0,954	0,834	0,928
p-value			0.287			0.524			0.265			0.126			0.495			0.082

NRS, numerical rating scale; SD, standard deviation;

**Table 2:** Pain scores (burning pain) in cohorts

	Burning pain Awake			Burning pain 10 min			Burning pain 20 min			Burning pain 30 min			Burning pain 40 min			Burning pain leaving		
cohort	S	L	Overall	S	L	Overall	S	L	Overall	S	L	Overall	S	L	Overall	S	L	Overall
N	13	15	28	13	15	28	13	15	28	13	15	28	2	3	5	13	15	28
mean	2,08	0,53	1,25	2,31	0,2	1,18	2,23	0	1,04	2,15	0	1	2,5	0	1	2,08	0	0,96
SD	0,862	0,743	1,11	0,751	0,414	1,219	0,725	0	1,232	0,801	0	1,217	0,707	0	1,414	0,76	0	1,17
p-value			< 0.001			< 0.001			< 0.001			< 0.001			0.007			< 0.001

SD, standard deviation;

### Opioids

In mean, patients in cohort S received 0.0504 mg/kg (0.0315–0.0693) piritramide, patients in cohort L received 0.0474 mg/kg (0.0337–0.0611). There was no significant difference between cohorts regarding dose of piritramide (p = 0.389). ANOVA analyses showed a significant correlation between NRS level and opioid received per kg (p < 0.001). The VRS score for burning pain showed no significant correlation with opioid use (p = 0.146).

### Follow-up

12 patients from cohort S and 14 patients from cohort L underwent follow-up examination one month after surgery: no significant differences between cohort S and cohort L were detected (p = 0.901 for best corrected visual acuity (BCVA), p = 0.419 for BCVA without limitation of visual acuity). Re-Bubbling was necessary in 2 of 13 patients (15.4%) in cohort S and in 2 of 15 patients (13.3%) in cohort L, after which graft attachment was 100% in both cohorts.

A clear corneal graft was present in 11 of 12 patients (91.7%) in cohort S and in 14 of 14 patients (100%) in cohort L.

## Discussion

In this monocentric, retrospective feasibility analysis, we found that a single local application of 2% lidocaine gel at end of surgery significantly reduced early postoperative burning pain sensation as measured by VRS in patients undergoing DMEK with corneal abrasion under general anesthesia (Fig. 1). A trend towards lower overall pain scores on the NRS (Fig. 2) and a trend towards lower intravenous opioid requirements were observed, although there was no significant difference. Surgical outcomes after one month showed no significant differences.

The early postoperative burning pain sensation seems to be an underestimated problem in patients undergoing DMEK with corneal abrasion under general anesthesia, as all patients in cohort S reported burning pain sensation. Remarkably, few patients reported burning pain to recovery room staff, even when asked about the overall pain using the NRS. In the authors’ clinical experience, a second question about burning pain was necessary to obtain this information, which the results of the present study suggest.

Even ocular surgery can lead to a relevant pain burden, although it is often considered to be less painful. [7–9] To the best of our knowledge there has been no study dealing with burning pain early after DMEK with corneal abrasion. DMEK is known as a surgical procedure causing relatively low pain levels according to sutureless procedure preserving eye surface integrity. [10–12] The pain scores after DMEK with corneal abrasion, especially the burning pain, seem to be due to the influence of nociceptors located only in the epithelial area of the corneal tissue. [13] In large corneal abrasions bandage soft contact lenses (BSCL) are part of the standard management at our clinic.

In the authors’ experience, patients’ well-being in the recovery room is severely affected by pain sensations after corneal abrasion. In addition, it may falsely suggest a painful IOP rise due to intracameral gas tamponade. [14] Standard treatment with intravenous opioids/non-opioids appears to be inadequate. Local administration of lidocaine gel at the end of the surgical procedure by the surgeon appears to significantly reduce the patient’s pain burden and has no effect on the surgical outcome. These results are consistent with previously published literature that has demonstrated successful treatment of patients with neuropathic pain with topical lidocaine for zoster ophthalmicus, diabetic peripheral neuropathy, carpal tunnel syndrome, or other causes. [15, 16]

With respect to the efficacy of the topical anesthesia three aspects have to be taken into account: (i) the number and function of corneal nerves shows significant interindividual variations [17], (ii) several corneal diseases [18] and surgical procedures at the eye - such as cataract surgery and DMEK [19] – lead to a reduced number and function of corneal nerves by cutting through them during corneal incisions and (iii) finally there are several systemic diseases affecting corneal nerve function, such as multiple sclerosis and several peripheral neuropathies [20–25]. All this shows that huge interindividual variations exist in corneal pain response. This even more underscores the significant effect of our findings here, but also calls for more prospective and disease-specific studies in the future.

The results of the present study suggest that patients undergoing corneal abrasion as part of DMEK have relevant pain sensations in the recovery room, although the literature shows that pain is low in the subsequent postoperative days. [3] Burning pain questions should be used in the pain assessment to capture these specific areas of pain sensation. A single dose of topical lidocaine gel at the end of surgery takes the burden of burning pain off the patient, without affecting the surgical outcome. Prospective clinical trials are needed to evaluate that discovery further.

### Limitations

The limited number of patients included is due to the research topic. The retrospective nature of this study causes all known limitations. Future prospective trials will have to analyze the effect of additional topical analgesia for the later postoperative period and the effects of early analgesia on later pain symptoms.

## Data Availability

All data are available upon reasonable request at the corresponding author.
